# Transaxillary Branch-to-Branch-to-Branch Carotid Catheterization Technique for Triple-Branch Arch Repair

**DOI:** 10.1177/15266028231169169

**Published:** 2023-05-24

**Authors:** Carlota F. Prendes, Paolo Spath, Jan Stana, Tarek Hamwi, Sven Peterss, Konstantinos Stavroulakis, Maximilian Pichlmaier, Nikolaos Tsilimparis

**Affiliations:** 1 Department of Vascular Surgery, Ludwig-Maximilians-University Hospital, Munich, Germany; 2 Department of Cardiac Surgery, University Hospital Munich, Ludwig Maximilian University, Munich, Bavaria, Germany

**Keywords:** aortic endovascular arch repair, percutaneous, axillary access, through-and-through guidewire, minimally invasive, innovation, branch

## Abstract

**Purpose:**

To describe the transaxillary branch-to-branch-to-branch carotid catheterization technique (tranaxillary 3BRA-CCE IT) for cannulation of all supra-aortic vessels using only 1 femoral and 1 axillary access during triple-branch arch repair.

**Technique:**

After deployment of the triple-branch arch device, catheterization and bridging of the innominate artery (IA) should be performed through a right axillary access (cutdown or percutaneous). Then, the retrograde left subclavian (LSA) branch should be catheterized (if not preloaded) from a percutaneous femoral access, and a 12×90Fr sheath should be advanced to the outside of the endograft. Subsequently, catheterization of the left common carotid artery (LCCA) antegrade branch should be performed, followed by snaring of a wire in the ascending aorta which was inserted through the axillary access, creating a branch-to-branch-to-branch through-and-through guidewire. Over the axillary access, a 12×45Fr sheath should be inserted into the IA branch and looped in the ascending aorta using a push-and-pull technique so that it faces the LCCA branch, allowing for stable catheterization of the LCCA. The retrograde LSA branch should then be bridged following the standard fashion.

**Conclusions:**

This series of 5 patients demonstrates that triple-branch arch repair can be performed with the transaxillary 3BRA-CCE IT, allowing catheterization of the supra-aortic vessels without manipulation of the carotid arteries.

**Clinical Impact:**

The transaxillary 3BRA-CCE IT allows catheterization and bridging of all supra-aortic vessels in triple-branch arch repair through only 2 vascular access points, the femoral artery and the right axillary artery. This technique avoids carotid surgical cutdown and manipulation during these procedures, reducing the risk of access site complications, including bleeding and reintervention, reintubation, cranial nerve lesions, increased operating time, and so on, and has the potential to change the current vascular access standard used during triple-branch arch repair.

## Introduction

Open surgery remains one of the best treatment strategies for aortic arch repair, with associated perioperative mortality and stroke rates of up to 8.8% and 7.7%, respectively.^1^ In unfit patients, however, these rates can increase up to 16% and 18%, respectively.^1^ Device innovation has allowed the creation of multiple endovascular aortic arch solutions, including fenestrated and branched custom-made devices, in situ laser fenestration, and surgeon-modified endografts.^1,2^ These minimally invasive techniques have demonstrated good postoperative outcomes, and triple-branch aortic arch repair has become a leading endovascular solution for these patients.^1,3^ However, despite comparably low combined stroke/mortality rates, it requires access to the supra-aortic vessels.^4^ Traditionally, this was performed through bilateral carotid, which allows distal carotid clamping during stent-graft deployment to avoid potential cerebral embolization and left brachial artery cutdown.^5^ However, surgical cutdown of the carotid arteries for branch cannulation and bridging stent-graft delivery increases the overall operating time of these procedures and can be associated with severe complications, such as hematoma requiring reintervention and reintubation, cranial nerve injuries, and iatrogenic carotid artery dissection.^5^ Alternative techniques that allow bypassing the need for surgical exposure of the supra-aortic vessels in patients undergoing triple-branch arch repair procedures appear reasonable. The transfemoral approach to cannulate the left carotid artery using steerable sheaths has been proposed, but this technique is limited by the length of the available steerable sheaths and the long distance from the femoral arteries, making steerability and stable cannulation challenging.^6^

We present a technique developed in our center, which enables cannulation of all supra-aortic vessels with a unique right axillary access and femoral access called the transaxillary branch-to-branch-to-branch carotid catheterization technique (transaxillary 3BRA-CCE IT).

## Case Report

### Patient Presentation

**Figure 1.**
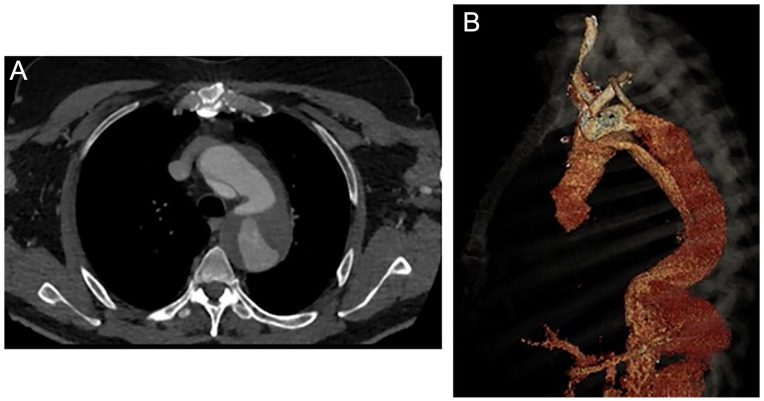
Preoperative computer tomography angiography reconstructions using a dedicated 3D workstation, showing (A) axial, and (B) 3D reconstruction of the 65-mm aortic arch and descending thoracic aorta aneurysm, with persistent false lumen perfusion and multiple entry tears at the level of the aortic arch and proximal descending aorta. As can be observed in the 3D reconstruction, the LSA is distally dissected and is perfused from both the true and false lumens.

A 61-year-old patient with a post-type-A dissection aortic arch and proximal thoracic descending aneurysm was evaluated in the outpatient clinic. He had undergone ascending aorta and hemiarch replacement with a 28-mm graft 2 years earlier. The computer tomography angiography (CTA) revealed a 65-mm aortic arch aneurysm, with persistent false lumen perfusion and multiple entry tears at the level of the arch and descending thoracic aorta. The innominate artery (IA) and left common carotid artery (LCCA) both arose from the true lumen, while the left subclavian artery (LSA) had perfusion from both the true and false lumens. (Figure 1A and B). Despite a young age, the patient presented significant comorbidities, including chronic obstructive pulmonary disease and obstructive sleep apnea syndrome, as well as prior sternotomy and aortic repair. The case was presented in an interdisciplinary aortic conference, and given the multiple comorbidities, prior aortic replacement surgery, and patient’s preference, an endovascular solution with a triple-inner-branch endograft was recommended. The patient signed an informed consent form for the procedure, as well as for clinical data collection and publication.

**Figure 2.**
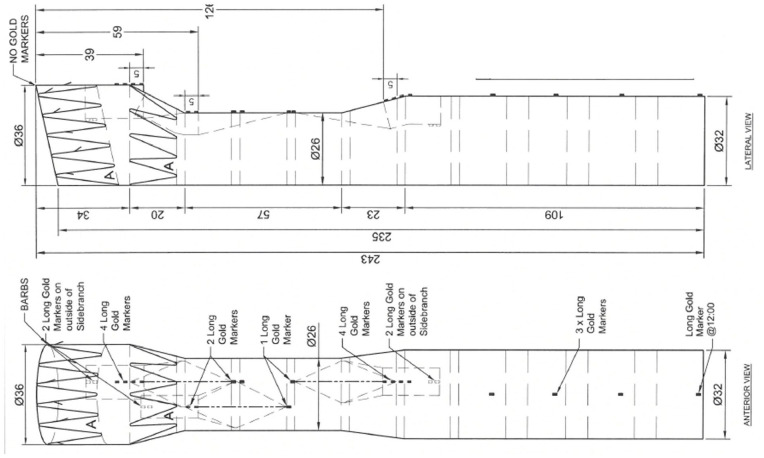
Preoperative graft plan showing the custom-made triple-inner-branch arch device with a proximal diameter of 36 mm, a distal diameter of 32 mm, and an overall length of 243 mm. The designed device presents 2 antegrade branches for the innominate artery and the left carotid artery and a preloaded retrograde branch for the left subclavian artery.

The patient presented a “healthy” proximal landing zone in Ishimaru zone 0, inside the prior ascending aortic graft. A custom-made triple-branch arch endograft (Cook Medical, Bloomington, Indiana) with a proximal diameter of 36 mm, a distal diameter of 32 mm, and an overall length of 243 mm (Figure 2) was planned. The branches for the IA and the LCCA were designed in antegrade fashion, while the LSA branch was a preloaded retrograde perfusion branch. Finally, a distal extension and false lumen occlusion using a thoracic endograft with a proximal diameter of 36 mm, distal diameter of 28 mm, and total length of 257 mm and 38-mm candy-plug device were planned.^7^

### Procedure

The procedure was conducted in an elective setting in a hybrid operating room (Artis zeego; Siemens Healthineers, Erlangen, Germany) under general anesthesia. The general procedure for implantation of a triple-inner-branch endograft has been previously described in detail.^8^

Percutaneous femoral access was performed using ultrasound-guided puncture. Two ProStyle percutaneous suture devices (Abbott, Plymouth, Minnesota) were placed on the right femoral artery, while a 4Fr sheath was inserted in the left femoral artery for angiographic and lower-body blood pressure control. Surgical exposure of the right axillary artery was obtained, and the patient was fully heparinized to achieve an activated clotting time of 250 to 300 seconds. A double-curved Lunderquist extra-stiff guidewire (Cook Medical) was advanced across the aortic valve into the left ventricle, and an angiographic catheter was brought to the ascending aorta over the right axillary artery.

The endograft was then advanced from the femoral access into the ascending aorta. The Munich Valsalva implantation technique (MuVIT)^9,10,11^ for cardiac output reduction was used to obtain a systolic blood pressure of 60 mm Hg. Once obtained, the endograft was fully deployed. Immediately thereafter, a double-curved Lunderquist guidewire was inserted into the preloaded catheter in the retrograde LSA branch, and the main endograft delivery system and the first Lunderquist guidewire used were retracted. A short 20Fr introducer sheath was advanced into the right femoral artery, and a 12×90Fr Flexor sheath (Cook Medical) was inserted over the newly placed Lunderquist guidewire.

**Figure 3.**
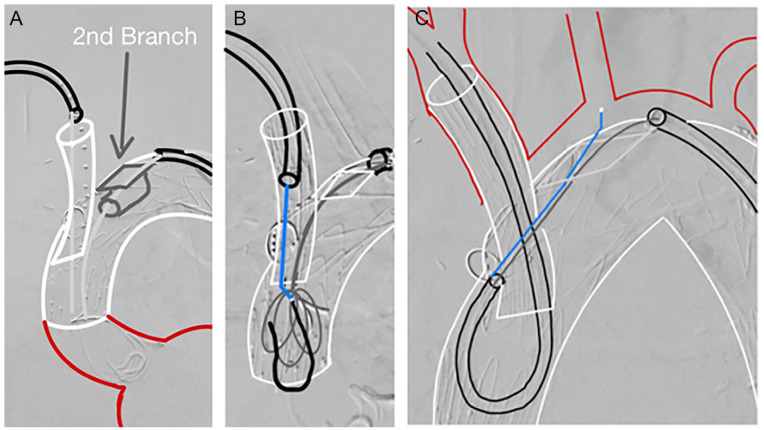
Schematic representation of the Munich branch-to-branch-to-branch through-and-through implantation technique (transaxillary 3BRA-CCE IT) using a (A) 12×45Fr sheath introduced through the axillary artery and a 12×90Fr introduced through the femoral artery and the retrograde precanulated branch. After catheterization of the second antegrade branch from the femoral access, (B) EN Snare was advanced to the ascending aorta through the femoral access, and a 400-mm Terumo guidewire through the axillary access, followed by (C) looping of the 12×45Fr axillary sheath through the first antegrade branch and into the second antegrade branch, with catheterization of the left common carotid artery with the aid of a puncture buddy wire and a Berenstein II catheter.

After complete endograft deployment, the first antegrade branch was catheterized through the axillary access. Correct branch cannulation was confirmed with retraction of an UF catheter, after which the first antegrade branch was bridged using a thoracic custom-made extension (Cook Medical) with a proximal diameter of 13 mm, a distal diameter of 16 mm, and a total length of 74 mm. The thoracic extension was exchanged for a 12×45Fr sheath, which was advanced through the axillary artery into the IA branch (Figure 3A). Then, the transfemoral 12×90Fr sheath was advanced through the LSA retrograde branch and positioned on the outside of the triple-branch arch device, through which the LCCA antegrade branch was cannulated with the aid of a Berenstein II catheter (Cordis, Miami Lakes, FL 33014) and a Terumo guidewire (Terumo, Leuven Belgium). After cannulation, the Terumo guidewire was exchanged for a stiff guidewire, which was then advanced, through the retrograde branch and the antegrade LCCA branch, into the ascending aorta (Figure 3B).

Over the axillary sheath, a 400-cm-long Terumo guidewire was advanced into the ascending aorta. Using an EN Snare (Merit Medical System, South Jordan, Utah), the 400-cm Terumo guidewire was snared, establishing a branch-to-branch-to-branch through-and-through guidewire (Figure 3B). The 12×45Fr right axillary sheath was advanced through the IA antegrade branch and looped using a “pull-and-push” technique (Figure 3C), forcing a change of direction of the sheath after the proximal end of the IA antegrade branch, allowing for orientation of the tip of the sheath toward the LCCA antegrade branch.^10^ A buddy puncture was then made on the axillary sheath, and a 7×55Fr sheath was advanced to the tip of the LCCA branch. Then, with the aid of a Berenstein II catheter and a 260-mm Terumo guidewire, cannulation of the LCCA was performed (Figure 3C). The hydrophilic wire was exchanged for a stiff Rosen wire (Cook Medical), and the 7×55Fr introducer sheath was advanced into the LCCA. The LCCA was then bridged with an 8×57-mm BeGraft Plus (Bentley InnoMed GmbH, Hechingen, Germany) using the bare-back technique. After angiographic control, the through-and-through guidewire is removed from the femoral artery (while maintaining the 12×90Fr sheath in place), and the IA docking zone was postdilated with a 12×20-mm balloon. At this point, through the 12×90Fr transfemoral sheath, catheterization of the LSA was performed with the aid of a Berenstein II catheter. First, a 10×37-mm BeGraft system was deployed proximally, extending the LSA branch, which was then extended distally into the LSA with a 13×50mm VIABAHN (W.L. Gore & Associates, Flagstaff, Arizona). Finally, to exclude false lumen perfusion, the endograft was extended distally with a 36×28×257-mm thoracic component (TX2; Cook Medical).

**Figure 4.**
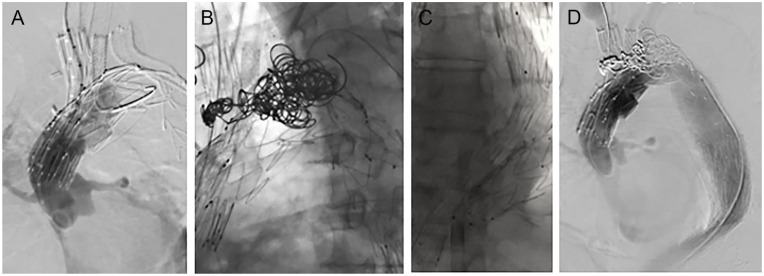
Intraoperative angiographic control showing (A) a type Ia endoleak secondary to kinking of the proximal endograft with bird-beaking, (B) coil embolization of proximal and distal parts of the aortic arch with 16 Nester coils, (C) distal extension with a thoracic device to the level of the celiac trunk and deployment of a 38-mm candy plug in the false lumen for false lumen perfusion, and (D) final angiographic control without residual false lumen perfusion and resolution of the type Ia endoleak.

Angiographic control revealed a type Ia endoleak secondary to partial kinking of the proximal part of the endograft, causing bird-beaking and persistent perfusion of the proximal and distal parts of the arch (Figure 4A). As proximal extension was not possible, the distal false lumen was cannulated, and coil embolization of the false lumen with 16 Nester coils (Cook Medical) was performed. Subsequent angiographic control showed resolution of the type Ia endoleak, without evidencing residual perfusion of the false lumen (Figure 4B). The procedure was then completed with deployment of a 38-mm candy plug in the false lumen (Figure 4C).

**Figure 5.**
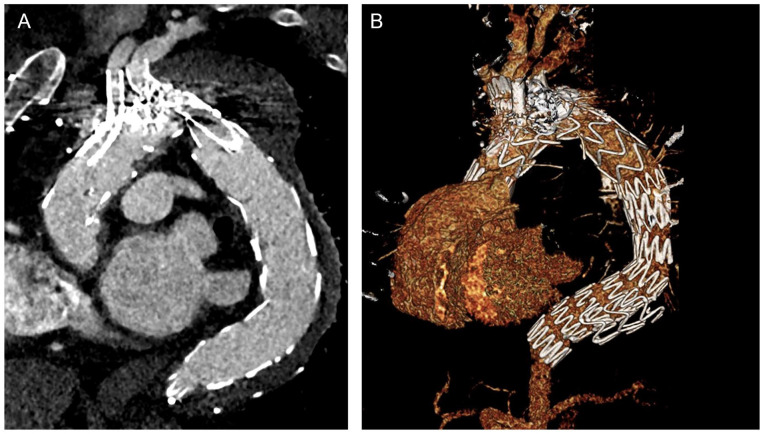
Six-month postoperative computer tomography angiography image showing the (A) sagittal plane and (B) the 3D reconstruction of the aortic arch, showcasing the graft with the supra-aortic vessels, the candy plug and complete thrombosis of the false lumen, and embolization of the arch.

The patient was extubated in the operating theater and presented no immediate neurological complications. Control CTA was performed on the seventh postoperative day, showing correct endograft deployment and perfusion of all supra-aortic vessels, and the patient was discharged on the eighth postoperative day in a good overall condition. The 7-month follow-up CTA showed a satisfactory result, with complete thrombosis of the false lumen proximally to the candy plug, and patency of all the supra-aortic vessels (Figure 5).

### Our Experience

Since March 2022, a total of 7 cases have been performed in our center with the transaxillary 3BRA-CCE technique. The 30-day mortality and 30-day neurologic complication rates were 0%. As reported in this technical note, there was 1 type Ia endoleak, treated with false lumen coil embolization, and 1 type Ic endoleak, requiring relining of the LCCA bridging stent. However, we do not believe that the type Ic endoleak had a direct relation to the 3BRA-CCE IT technique, rather with insufficient oversizing of the LCCA bridging stent. No other type Ia or III endoleaks were observed. The postoperative evolution of the patients was satisfactory, and the control CTAs showed correct positioning of the endografts, with patent supra-aortic vessels, and no signs of distal embolization (Table 1).

**Table 1.** Demographic characteristics and procedural data of the seven cases performed with the transaxillary 3BRA-CCE technique.

Case #	Age, sex	Pathology	Repair/concomitant procedures	Total OP time (hours)	Discharge (postoperative day)	30-Day mortality	30-Day stroke	Reinterventions	FU time (months)
1	61 years, male	Post-TAAD 65-mm aortic arch aneurysm	3× bTEVAR + distal extension + candy plug + coiling of the false lumen	5:49	8	No	No	No	11
2	64 years, male	Post-TAAD 57-mm aortic arch aneurysm	3× bTEVAR + distal extension	4:23	6	No	No	Yes, relining of the left common carotid artery stent due to small dimension, 4 months PO	7
3	85 years, male	Ruptured 37-mm aortic arch PAU	3× bTEVAR	3:12	35	No	No	Thoracic tube, VATS	7
4	68 years, male	Post-TAAD 60-mm aortic arch aneurysm and 72-mm thoracic aneurysm	3× bTEVAR + distal extension	5:00	16	No	Delirium, microemboli in control MRT	No	7
5	78 years, male	Post-TAAD 62-mm aortic arch and thoracic descending aneurysm	3× bTEVAR + distal extension	5:32	10	No	No	No	5
6	73 years, male	PAU of the ascending aorta	3× bTEVAR	3:42	22	No	No	No	3
7	84 years, male	Symptomatic aortic arch aneurysm with a max. diameter of 65 mm	3× bTEVAR + distal extension	4:15	21	No	No	No	1

OP = operating time, FU = follow-up, TAAD = type A aortic dissection, PO = postoperative, PAU = penetrating aortic ulcer, VATS = video assisted thoracic surgery, bTEVAR = branched thoracic endovascular aortic repair, MRT = magnetic resonance tomography.

## Discussion

To our knowledge, this is the first report of total endovascular arch repair with a triple-inner-branch device using the transaxillary 3BRA-CCE IT for the cannulation of all supra-aortic vessels, thus avoiding carotid artery exposure and additional manipulation.

Open surgical repair of degenerative aortic arch aneurysms in patients with high surgical risk or requiring re-do surgery can be associated with significant perioperative morbi-mortality rates.^1^ In these patients and in experienced centers, endovascular repair of the arch with either fenestrated or branched devices has demonstrated excellent results, with overall mortality rates of 3.7% and 5% and overall stroke rates of 6% and 5% for fenestrated and branched devices, respectively.^6,12^ Similarly, promising results have been reported for endovascular repair of post-type-A aortic arch aneurysms, which has been associated with a combined mortality/stroke rate of 4%.^4^

However, despite significant advances made in total endovascular aortic arch repair, arch branch devices traditionally require surgical cutdown of the carotid arteries for cannulation and direct arterial control, preventing possible intracranial embolization during manipulation and bridging stent deployment. Although carotid artery exposure is, a priori, technically not too complex, it increases the total intervention time and can be associated to complications. Konstantinou et al found a 9% rate of peripheral neurological dysfunction following debranching of the supra-aortic vessels before endovascular repair, as well as access-related complications being the most prevalent cause of reinterventions in these patients.^7^ Furthermore, there will be a subset of patients presenting hostile necks (prior surgical intervention, radiation therapy, very short necks, and so on) in whom carotid surgical cutdown may be one of the key challenges of the procedure. The aim of this technique was, therefore, to make arch branch procedures more minimally invasive, avoiding surgical cutdown and additional manipulation of the supra-aortic vessels, decreasing the risk of nerve injury, reinterventions, access site complications including bleeding and dissections, as well total intraoperative time. Another benefit is that it also avoids the need for carotid puncture, which, especially in postdissection patients, can lead to dissection of the puncture site and need for carotid clamping and repair. The transaxillary 3BRA-CCE IT allows these procedures to be accomplished through a percutaneous femoral access and a minimal axillary incision. Furthermore, this technique could be combined with a percutaneous access of the axillary artery, which has proven to have excellent results, thus achieving a total percutaneous arch branch repair.^13^

In our experience, the absence of embolic protection during carotid stent delivery appears safe. There were no cases of postoperative stroke in the 7 patients presented in this series, with 1 patient presenting microemboli in a control magnetic resonance tomography, however, without clinical correlation. No embolic protection filters were used, and we would not recommend routine use of these. As the through-and-through technique is performed inside the deployed endograft, we believe that this only minimally increases arch manipulation. However, until more experience is gathered, it would be advisable to avoid performing the 3BRA-CCE IT in patients deemed at high risk of distal embolization, such as patients with a shaggy aorta or plaque burden in the common carotid arteries. In addition, patients with postdissecting aneurysms and dissected carotid arteries should preferably undergo exposure of the left LCCA.

This technique was carried out with pull-and-push maneuvering of the axillary 12×45Fr sheath, as previously described by Mougin et al,^6^ wherein they used this technique for LCCA cannulation from the femoral arteries. However, in contrast to the methods of Mougin et al,^6^ we used the pull-and-push maneuver to establish a branch-to-branch-to-branch connection, passing a wire through the retrograde LSA branch to the outside of the endograft, then into the antegrade LCCA branch, and then snaring it from the antegrade IA branch inside the endograft, to achieve more stability. In comparison to a transfemoral percutaneous access, our technique allows for increased control and manipulation of the guidewires and catheters given the proximity of the target vessels to the access point. Although this is the first report in the literature describing the use of the transaxillary 3BRA-CCE IT, the use of a branch-to-branch through-and-through guidewire was previously described in an emergent triple-branch arch case to accommodate rotation error.^14^,^15^

In our case series, snaring and establishment of a through-and-through guidewire were always performed in the proximal part of the aortic graft, avoiding unintended manipulation inside the ascending aorta, reducing the risk of cerebral or distal embolization. Additionally, the proximal part of the triple-branch endograft has sufficient space for snaring the guidewire. The establishment of a branch-to-branch-to-branch through-and-through guidewire allows steering of the 12×45Fr sheath into the aortic arch and second antegrade branch, allowing sufficient stability to be achieved for advancement of a 7×55Fr sheath or a 9Fr stent into the LCCA. In comparison to a femoral through-and-through wire, this technique gives more stability and maneuverability given the shorter length of the working materials. Finally, in our experience, the optimal outcomes with this procedure can be obtained with the use of a Flexor sheath (Cook Medical) which combines flexibility and resistance, whereas the use of steerable sheaths may be limited by a weaker structure and high risk of rupture of the expensive device.

**Figure 6.**
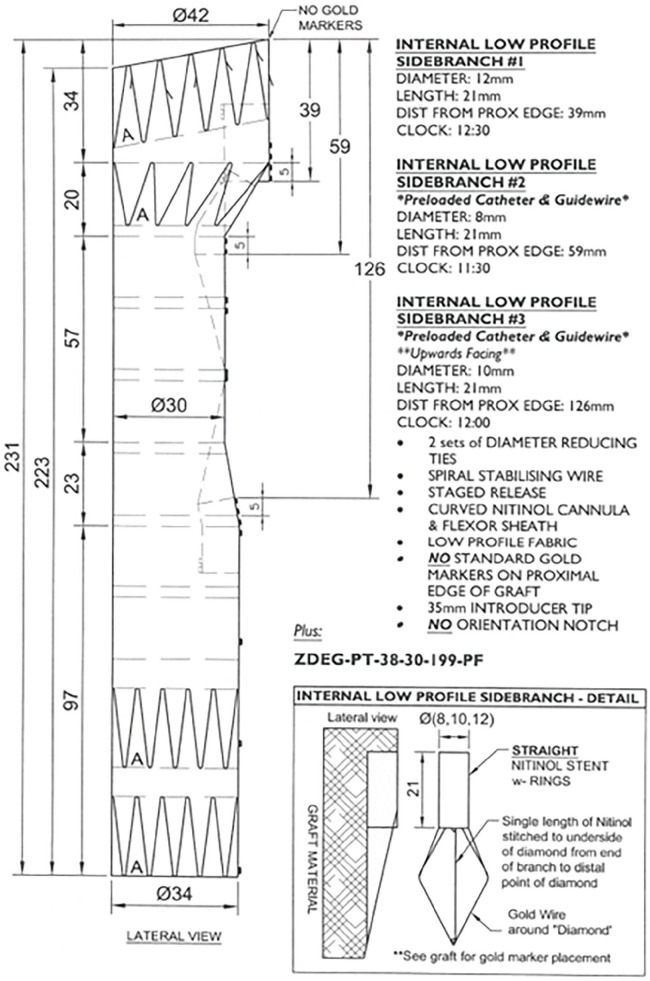
Example of a triple-branch arch graft plan with a preloaded catheter and guidewire in the retrograde LSA branch and in the antegrade left common carotid artery branch.

Although this technique has, of yet, only been performed in 7 patients, this has become our primary approach for LCCA branch catheterization in arch-branched procedures, and despite the small number of patients, it appears to be safe and feasible, not increasing the risk of perioperative stroke or mortality rates. Finally, current triple-branch arch plans can be performed with a preloaded wire from the LSA into the LCCA branch, facilitating LCCA cannulation and the transaxillary 3BRA-CCE IT (Figure 6).

## Conclusion

To our knowledge, this technical note is the first report of aortic arch repair with a triple-branch aortic arch endograft using transaxillary 3BRA-CCE IT for successful cannulation and bridging stent deployment in all supra-aortic trunks, using only an axillary and a femoral access. Although only 5 cases have been included, this technique appears to be safe and effective in experienced centers.
